# Screening and validation of ZFYVE27 as a potential diagnostic biomarker for osteoporosis via integrative bioinformatics and machine learning approaches

**DOI:** 10.3389/fimmu.2026.1862140

**Published:** 2026-06-24

**Authors:** Libo Zhou, Zirui Liu, Zhongcheng Liu, Lei Wen, Maoqiang Lin, Bin Geng, Yayi Xia

**Affiliations:** 1 Department of Orthopaedics, Lanzhou University Second Hospital, Lanzhou University, Lanzhou, China; 2 Orthopaedic Clinical Research Center of Gansu, Lanzhou, China; 3 Intelligent Orthopaedic Industry Technology Center of Gansu Province, Lanzhou, China

**Keywords:** bioinformatics analysis, immune cell, inflammatory microenvironment, machine learning, osteoimmunology

## Abstract

**Purpose:**

Osteoporosis (OP) is a systemic metabolic skeletal disorder characterized by diminished bone mineral density, deteriorated bone microarchitecture, and a consequently heightened susceptibility to fragility fractures. Bioinformatics approaches serve as a crucial bridge between genomic investigations and clinical translation, and have been extensively utilized in OP research. Nevertheless, the precise identification of core pathogenic genes and the subsequent development of robust and accurate diagnostic biomarkers remain urgent clinical imperatives.

**Methods:**

Initially, we identified differentially expressed genes (DEGs) by comparing transcriptomic profiles between healthy controls and osteoporotic patients, followed by functional enrichment analyses of associated biological processes and signaling pathways. Weighted gene co-expression network analysis was subsequently applied to isolate disease-specific module genes. By intersecting these module genes with the DEGs, OP-related DEGs were precisely delineated. The LASSO regression algorithm was utilized to filter seven hub candidate genes. Subsequently, Support Vector Machine and Random Forest machine learning algorithms were employed to further optimize and cross-validate these potential diagnostic biomarkers. The intersection of these multi-algorithmic outputs ultimately designated the core biomarker. Furthermore, an ovariectomized (OVX) mouse model of OP was established to experimentally validate ZFYVE27 expression levels. A competitive endogenous RNA regulatory network was concurrently constructed to elucidate its post-transcriptional regulatory mechanisms underlying OP pathogenesis.

**Results:**

ZFYVE27 emerged and was successfully validated as an optimal diagnostic biomarker for OP. Compared to the sham-operated group, both mRNA and protein expression levels of ZFYVE27 were significantly upregulated in the OVX model group (*p* < 0.01), strongly suggesting that ZFYVE27 plays a critical role in the pathological progression of OP.

**Conclusion:**

The present study identifies ZFYVE27 as a novel biomarker for the clinical diagnosis of OP. Furthermore, our findings provide a solid theoretical foundation for further elucidating the molecular pathogenesis of OP and developing targeted therapeutic interventions.

## 1 Introduction

Osteoporosis (OP) is a skeletal disorder characterized by reduced bone mineral density and bone mass resulting from multiple factors, accompanied by an elevated fracture risk, and is particularly prevalent in postmenopausal women (1). Postmenopausal osteoporosis (PMOP) is primarily driven by estrogen deficiency, which promotes the differentiation and activation of osteoclasts, thereby accelerating bone resorption to a rate that outpaces bone formation. This leads to substantial bone loss, especially during the perimenopausal period (2). Currently, treatments for OP include exercise, vitamin and mineral supplements, and pharmacological therapies (3). However, prolonged pharmacological treatments often exhibit plateauing efficacy and may elicit a spectrum of severe adverse effects (4). Recently, as research into pathogenic molecules has continually deepened and high-throughput sequencing technology has advanced, gene therapy has offered a fresh perspective on the treatment of OP. However, the primary molecular biological mechanisms underlying OP remain unclear. Therefore, identifying new molecular biomarkers and potential targets for drug development are crucial for OP.

The integration of bioinformatics and machine learning enables the synthesis of multi-omics data, facilitating the discovery of underlying patterns and multidimensional correlations (5, 6). This study analyzed gene expression data to identify differentially expressed genes (DEGs) between OP and control groups, followed by kyoto encyclopedia of genes and genomes (KEGG)/gene ontology (GO) functional and pathway enrichment analyses. Weighted gene co-expression network analysis (WGCNA) was applied to construct co-expression networks and extract OP-associated module genes. The intersection of DEGs with module genes yielded disease-related DEGs, which were further screened using machine learning algorithms. ZFYVE27 was identified as a potential biomarker for osteoporosis diagnosis, and its expression level was validated in the OP model. Cross-dataset validation, along with functional explorations of its interacting partners (GDPD3 and MARCO), further underscored the pivotal role of ZFYVE27 in OP pathogenesis. Furthermore, a construction of competitive endogenous RNA (ceRNA) network was constructed based on ZFYVE27.

## 2 Materials and methods

### 2.1 Data sources

The GSE35956 and GSE230665 datasets were downloaded from the gene expression omnibus (GEO, https://www.ncbi.nlm.nih.gov) database. The datasets were merged utilizing the “rbind” function in R, and batch effects were systematically eliminated using the “ComBat” algorithm. After merging and normalization, we finally obtained the expression matrices for 8 healthy controls and 17 patients with osteoporosis. We then used the “limma” and “sva” R packages to identify differentially expressed genes between the two datasets.

### 2.2 Differential analysis and gene enrichment analysis

The limma R package was used to perform differential analysis on the samples. DEGs were defined based on the threshold criteria of |log_2_FC| > 1 and an adjusted *p*-value < 0.05. Gene set enrichment analysis (GSEA) analysis was employed to assess pathway enrichment between high and low expression groups.

### 2.3 Functional analysis

GO and KEGG analyses compared cellular components, molecular functions, biological processes, and pathways between groups. CIBERSORT evaluated 22 immune cell infiltrations. GSEA explored links between gene sets, immune status, KEGG pathways, immune cell infiltration, and core genes.

### 2.4 WGCNA

WGCNA was employed to identify co-expressed gene modules associated with specific clinical traits. Initially, the topological overlap and connectivity of all genes were evaluated, followed by the construction of an adjacency matrix to quantify node correlation strengths. Next, the adjacency matrix was converted into a topological overlap matrix to quantitatively describe the similarity between nodes and the optimal value was determined. Outliers were also processed. Finally, through hierarchical clustering with a minimum of 50 genes, key modules were determined. The dynamic tree cut method was used for identifying modules, with the MEDissThres parameter set to 0.25.

### 2.5 Core gene screening based on machine learning

Using differentially expressed genes and WGCNA module genes, the disease-related differentially expressed genes were obtained. Three machine learning algorithms, namely least absolute shrinkage and selection operator (LASSO) regression, support vector machine recursive feature elimination (SVM-RFE), and random forest (RF), were then applied to screen key genes in osteoporosis. The candidate core genes were further determined by intersecting the gene sets selected by these algorithms. Finally, the expression levels of the identified core genes were validated in an independent external dataset.

Three distinct machine learning algorithms, RF, SVM-RFE, and LASSO regression, were employed to identify robust diagnostic biomarkers. For the RF model, the parameters were configured as follows: the number of decision trees was set to 500, the maximum depth of each tree was not explicitly restricted, the line width parameter was set to 2, and optionTrees was set to 65. For the SVM-RFE algorithm, 10-fold cross-validation (k = 10) was used for internal cross-validation during the recursive feature elimination process. When the number of remaining features exceeded 100, half of the features were removed in each iteration (halve. above = 100). The external cross-validation used to evaluate feature stability was also set to 10-fold (nfold = 10). The top 40 features were selected as the input for the SVM classifier. The SVM model was trained with the default kernel function (radial basis function) and other default parameters. For Lasso regression, the parameters were set as follows: family = “binomial”, nlambda = 100, alpha = 1, nfolds = 10.

### 2.6 Animals

In this study, twenty female wild-type C57BL/6J mice (18-20g, 8 weeks old) were purchased from the Medical Experimental Center of Lanzhou University (Lanzhou, China) and randomly divided into an ovariectomized (OVX) group and a sham group. Mice were housed under specific pathogen free conditions (22 °C, 12-h light/dark cycle). Prior to surgery, all mice were allowed a 7-day acclimation period. An OVX-induced OP model was established based on the previous description (7). Briefly, mice were anesthetized via intraperitoneal injection of pentobarbital sodium (50 mg/kg) and secured in a prone position. Bilateral ovariectomies were performed by exposing and excising the ovaries, after which the incisions were sutured. In the sham group, both ovaries were also exposed and all fatty tissues around them were removed, but the ovaries themselves remained intact. The experimental protocols were approved by the Laboratory Animal Welfare and Ethics Committee of Lanzhou University Second Hospital (D2024-964). All mice were handled following the Guide for the Care and Use of the Laboratory Animals of the National Institutes of Health. Two months postoperatively, all mice were euthanized via intraperitoneal injection of pentobarbital sodium (150 mg/kg). The femurs were collected for further micro-computed tomography (micro-CT) analysis, reverse transcription quantitative polymerase chain reaction (RT-qPCR), and western blotting assays.

### 2.7 micro-CT analysis

Mouse femurs were fixed with 4% paraformaldehyde solution at room temperature for 2 days and later scanned with a micro-CT scanner (Cat#SkyScan 1275, Aartselaar, Belgium) with the X-ray energy at 60 µA/50 kV at a resolution of 9 µm. Subsequently, a region of interest (ROI) spanning 0.5 mm below the growth plate was selected for the qualitative and quantitative evaluation of trabecular bone microarchitecture. The bone morphometric parameters used in the analyses included bone mineral density (BMD), trabecular bone volume per total volume (BV/TV), trabecular number (Tb.N), and trabecular separation (Tb.Sp).

### 2.8 RNA extraction and qPCR analysis

We extracted total RNA from mouse femurs using a SteadyPure Universal RNA Extraction Kit (Cat#AG21017, Accurate Biology, China) according to the manufacturer’s instructions. cDNA was synthesized by reverse transcription using the Evo M-MLV Mix Kit with gDNA Clean for qPCR (Cat#AG11728, Accurate Biology, China). Real-time quantitative polymerase chain reaction (RT-qPCR) was conducted using cDNA, primers, RNase-free water, and SYBR Green Premix Pro Taq HS qPCR (Cat# AG11718, Accurate Biology, China). GAPDH was used as an internal reference gene to normalize target gene expression, and the relative mRNA expression levels were calculated using the 2^−ΔΔCt^ method. The primer sequences for RT-qPCR (Tsingke Biotechnology, Beijing, China) are shown in **Supplementary Table 1**.

### 2.9 Western blotting

Total protein was extracted using RIPA lysis buffer (Cat# P0038, Beyotime Bio, Shanghai, China). Tissue lysates were collected and centrifuged at 12,000 rpm for 30 min. Protein concentration was determined using the BCA protein assay kit (Cat#P0009, Beyotime, Shanghai, China). Protein samples were mixed with loading buffer at the specified ratio and then heated at 100 °C for 10 minutes. Load 20-30 μg of protein onto a 10% SDS-PAGE gel. The gel was run at 90 V for 2 h, and then the protein was transferred to the PVDF membranes (Cat# IPVH00010, Millipore, USA). The membranes were blocked with 5% BSA for 1h at 4 °C, followed by overnight incubation at 4 °C with the following primary antibodies: ZFYVE27 (Cat#ab314323, Abcam, UK, 1:1,000) and GAPDH (Cat#YN5585, immunoway, USA, 1:5,000). On the following day, the membranes were incubated for 1 h with an HRP-conjugated goat anti-rabbit IgG secondary antibody (Cat#SA00001-2, Proteintech, USA, 1:10,000).

### 2.10 Construction of ceRNA networks

To reveal the potential post-transcriptional regulatory mechanisms of the three core genes, the lncRNA-miRNA-mRNA regulatory network was constructed. To be specific, microRNAs (miRNAs) targeting the three core genes were predicted according to miRanda, miRDB, miRWalk and TargetScan databases, respectively, and later miRNAs with scores of 1 in the four databases were screened.

### 2.11 Statistical analysis

Statistical analysis was performed using R software version 4.2.0 (available at http://www.r-project.org). The Wilcoxon test was used for assessing differences in continuous data between two subgroups. The Pearson correlation coefficient was applied to evaluate the relationship between two variables. All data are presented as mean ± SD, and were analyzed and plotted using GraphPad Prism 8.0 software (GraphPad Software, CA, USA). The normality of the data was evaluated by the Shapiro-Wilk test. For normally distributed data, statistical analysis was conducted using an unpaired t-test to assess intergroup differences. For non-normally distributed data, the Mann-Whitney U test or Kruskal-Wallis H test was applied to analyze intergroup differences. *p* values were indicated in the figure legends, and a *p* value < 0.05 was considered statistically significant. All experiments were performed at least three times independently.

## 3 Results

### 3.1 Functional enrichment analysis implicates calcium secretion and bone metabolism in OP pathology

**Figure 1:**
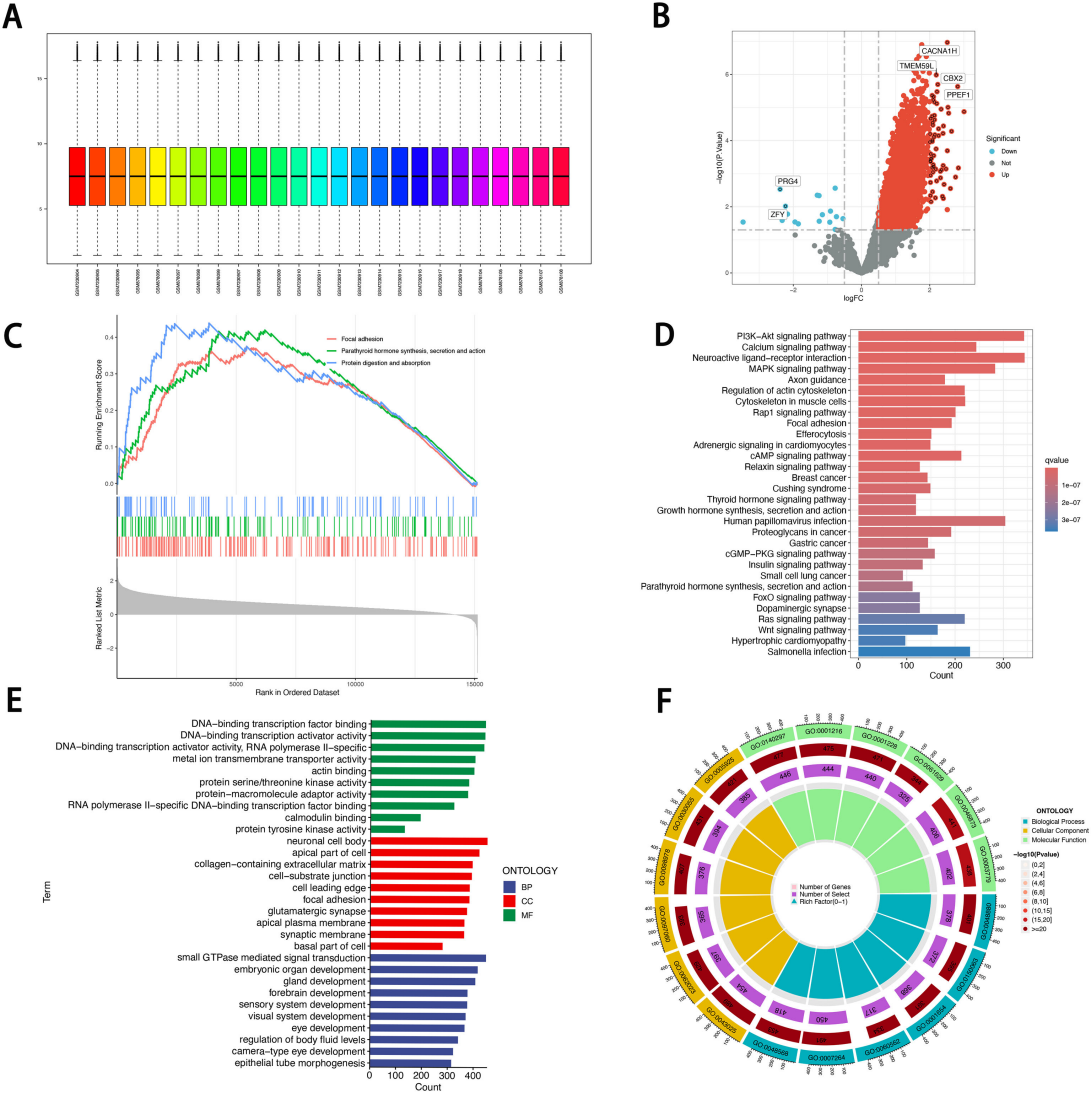
Identification and analysis of differential genes in the training set. **(A)** Data normalization. **(B)** Volcano plot showing differences between osteoporotic and healthy samples. **(C)** Upregulated pathways in GSEA. **(D)** KEGG analysis. **(E, F)** GO analysis. KEGG, kyoto encyclopedia of genes genomes; GO, gene ontology; GSEA, gene set enrichment analysis.

After standardizing the samples, differential analysis was conducted to identify 2,306 differentially expressed genes between osteoporotic and healthy samples (**Figures 1A, B**). Subsequent GSEA results revealed significant up-regulation of focal adhesions (FAs), parathyroid hormone (PTH) synthesis and secretion, and protein digestion and absorption (**Figure 1C**). KEGG analysis indicated the activation of adrenergic signaling in cardiomyocytes and PTH pathways (**Figure 1D**). Furthermore, GO analysis indicated that the differential genes were associated with protein serine/threonine kinase, actin binding and calmodulin binding in molecular functions category (**Figures 1E, F**). Differential genes were associated with neuronal cell bodies, collagen-containing extracellular matrix, and FAs in the cellular component category. They were related to small GTPase-mediated signal transduction, embryonic organ development, and sensory system development in the BP category.

### 3.2 OP-related gene sets identified by WGCNA

A scale-free gene co-expression network was constructed to identify OP-associated key modules. A power of β = 10 (scale-free R² = 0.9) was selected as the soft-thresholding parameter to generate a hierarchical clustering tree (**Supplementary Figures 1A–E**). Then, seven gene modules, each containing over 50 genes, were identified through hierarchical clustering and assigned different colors (**Supplementary Figures 1F–H**). The darkturquoise module with the highest correlation coefficient (R = 0.62) and 1,467 genes was selected for further analysis as the disease gene module.

**Figure 2:**
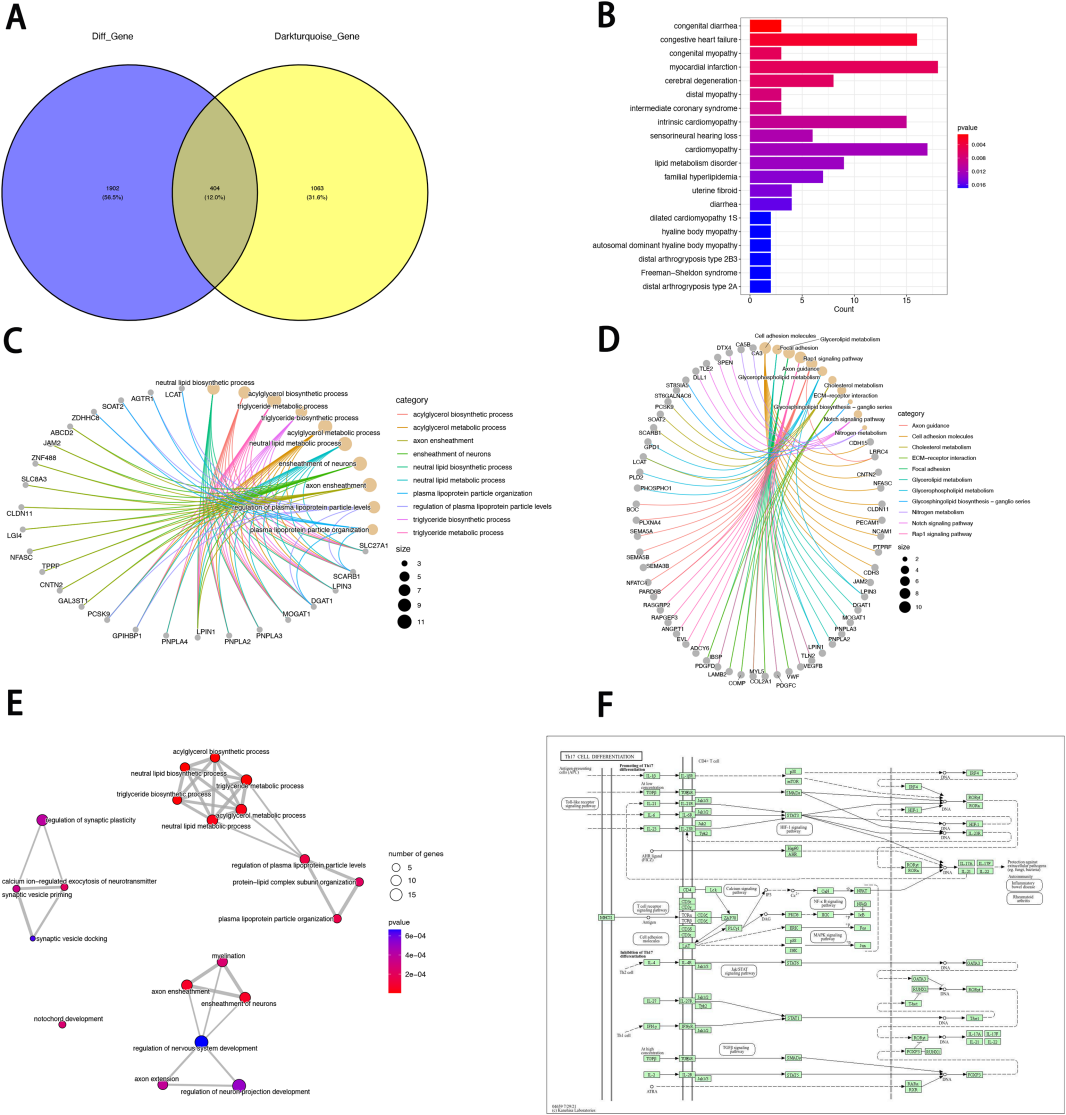
Analysis of differential gene function. **(A)** Intersection of differential genes and WGCNA module genes. **(B)** Disease enrichment analysis of intersection genes. **(C)** GO analysis of intersection genes. **(D-F)** KEGG analysis of intersection genes. WGCNA, weighted gene co-expression network analysis.

The intersection of differential genes (2,306) and module genes (1,467) yielded 404 disease-related differential genes (**Figure 2A**). Disease Ontology analysis of these genes suggested that metabolic diseases, such as lipid metabolism disorders and familial hyperlipidemia, might affect bone health (**Figure 2B**). Functional enrichment analysis indicated that the biosynthesis and metabolic processes of glycerides and neutral lipids played vital roles in maintaining bone health (**Figure 2C**). Glycerolipid, glycerophospholipid, and nitrogen metabolism pathways may influence osteoporosis progression through bone cell proliferation/differentiation mechanisms (**Figure 2D**). Pathway-specific gene networks were illustrated (**Figures 2E, F**), highlighting lipid components’ dual roles in membrane structure and cellular signaling.

### 3.3 LASSO regression analysis identifies seven core genes

**Figure 3:**
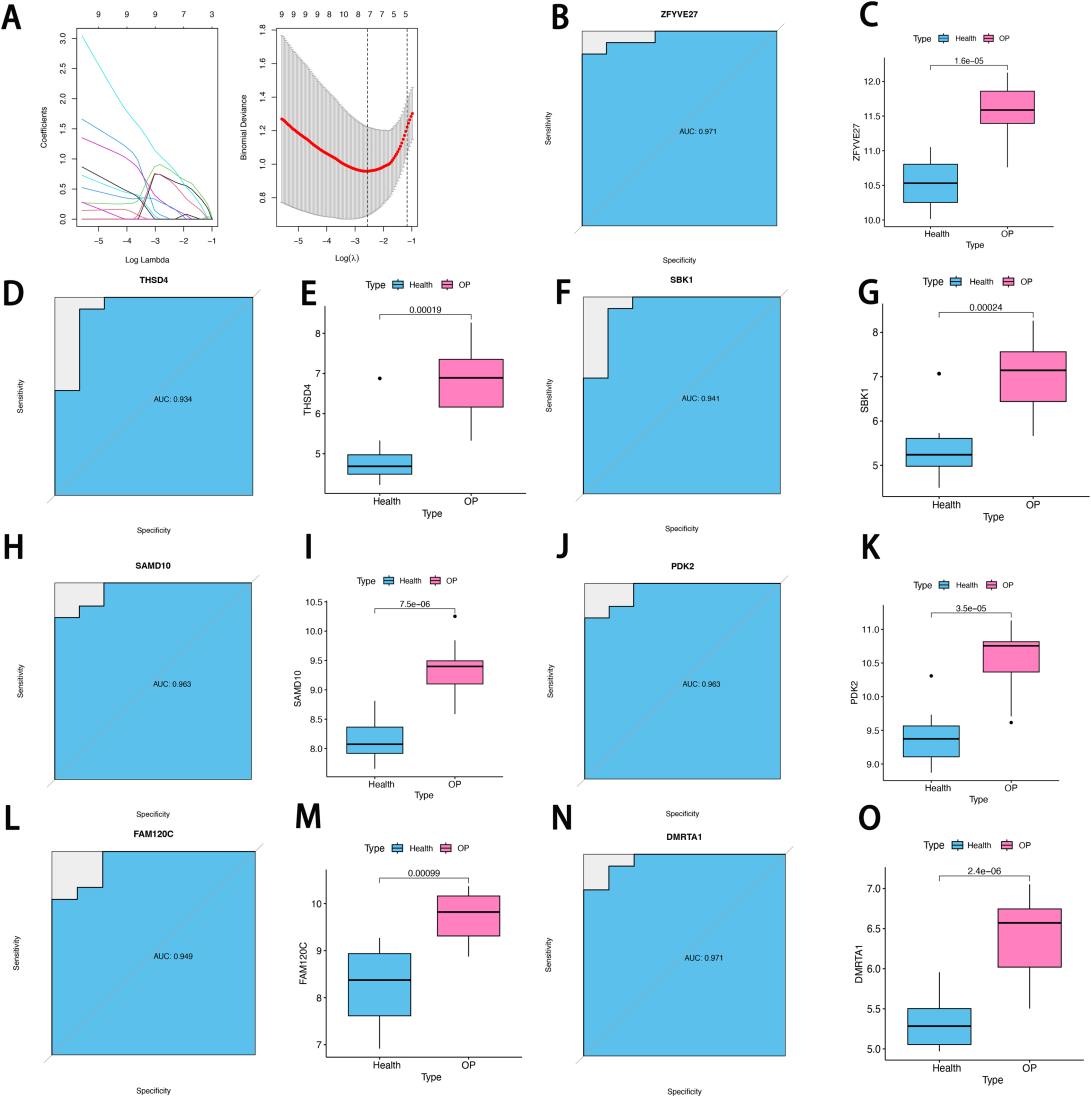
Lasso regression analysis to obtain key genes. **(A)** Identification of key genes using LASSO regression. **(B, C)** ROC curve and expression difference for evaluating the diagnostic performance of the signature gene (ZFYVE27). **(D, E)** ROC curve and expression difference for evaluating the diagnostic performance of the signature gene (THSD4). **(F, G)** ROC curve and expression difference for evaluating the diagnostic performance of the signature gene (SBK1). **(H, I)** ROC curve and expression difference for evaluating the diagnostic performance of the signature gene (SAMD10). **(J, K)** ROC curve and expression difference for evaluating the diagnostic performance of the signature gene (PDK2). **(L, M)** ROC curve and expression difference for evaluating the diagnostic performance of the signature gene (FAM120C). **(N, O)** ROC curve and expression difference for evaluating the diagnostic performance of the signature gene (DMRTA1). LASSO, least absolute shrinkage and selection operator; ROC, receiver operating characteristic; AUC, area under curve.

LASSO regression analysis was conducted on the disease-related differential genes (**Figure 3A**), resulting in the selection of FAM120C, THSD4, SAMD10, PDK2, ZFYVE27, SBK1, and DMRTA1 as core genes. To validate the expression and diagnostic prediction of these core genes in OP, receiver operating characteristic (ROC) curves were generated. The ROC curve validation showed that the combination of these seven genes had high diagnostic efficacy for OP (Area under curve > 0.9) and were highly expressed in the osteoporotic group compared with the healthy group (**Figures 3B–O**).

### 3.4 Core gene analysis

There were significant differences in multiple immune cell types between healthy and OP samples (**Supplementary Figures 2A–C**). T cell subsets, particularly CD4^+^ T cells, showed elevated activity in OP, suggesting enhanced functional or transcriptional states compared to those in healthy samples. Plasma cell scores were also higher in OP, underscoring B cells’ role in OP pathogenesis. Neutrophil activity was notably elevated in OP, a phenomenon potentially driven by estrogen deficiency, which provokes the secretion of chemokines and cytokines, thereby disrupting the balance between osteoblasts and osteoclasts. Natural killer cells exhibited increased activity, possibly linked to OP-associated inflammation. Gene set analyses revealed significant correlations with the Wnt and Notch signaling pathways (**Supplementary Figures 2D, E**), highlighting their involvement in OP mechanisms. These findings collectively emphasize dysregulated immune cell dynamics and pathway interactions in OP progression.

### 3.5 Machine learning combination identifies ZFYVE27 as a diagnostic marker

**Figure 4:**
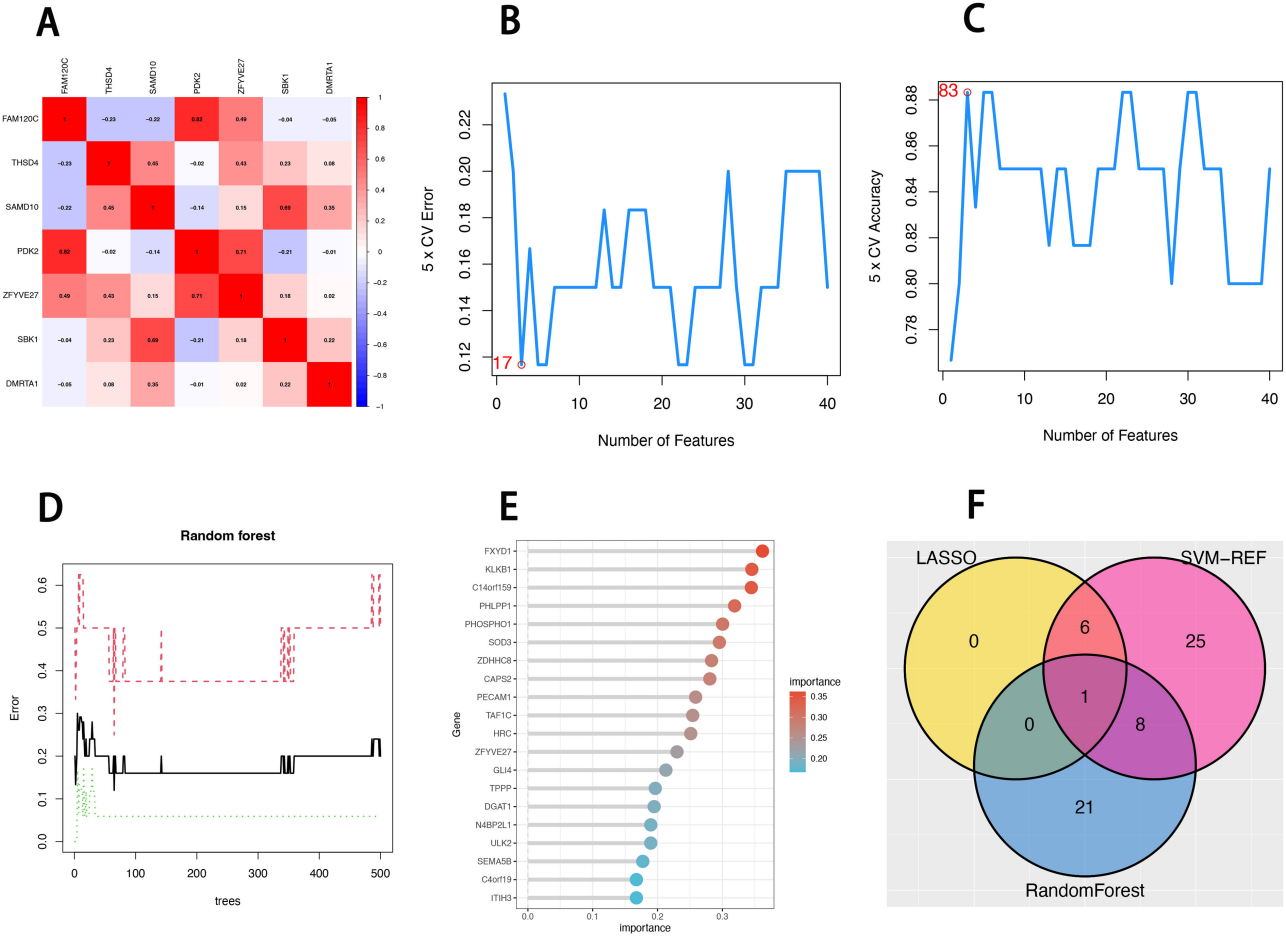
Machine learning combination analysis to identify core genes. **(A)** Correlation heatmap of key genes on the basis of LASSO. **(B, C)** Feature gene selection using the SVM-RFE algorithm. **(D)** RF algorithm showing the error rate for the osteoporotic group and the control group. **(E)** Genes ranked based on importance scores. **(F)** Venn diagram displaying the common feature genes identified by LASSO, SVM-RFE, and RF algorithms. SVM-RFE support vector machine recursive feature elimination; RF, random forest.

Lasso regression identified seven core genes (**Figure 4A**). Support vector machine (SVM) and RF analyses revealed 40 and 30 OP-related genes, respectively (**Figures 4B–E**), with the intersecting gene ZFYVE27 emerging as the diagnostic marker (**Figure 4F**).

### 3.6 Exploration of ZFYVE27 in OP

**Figure 5:**
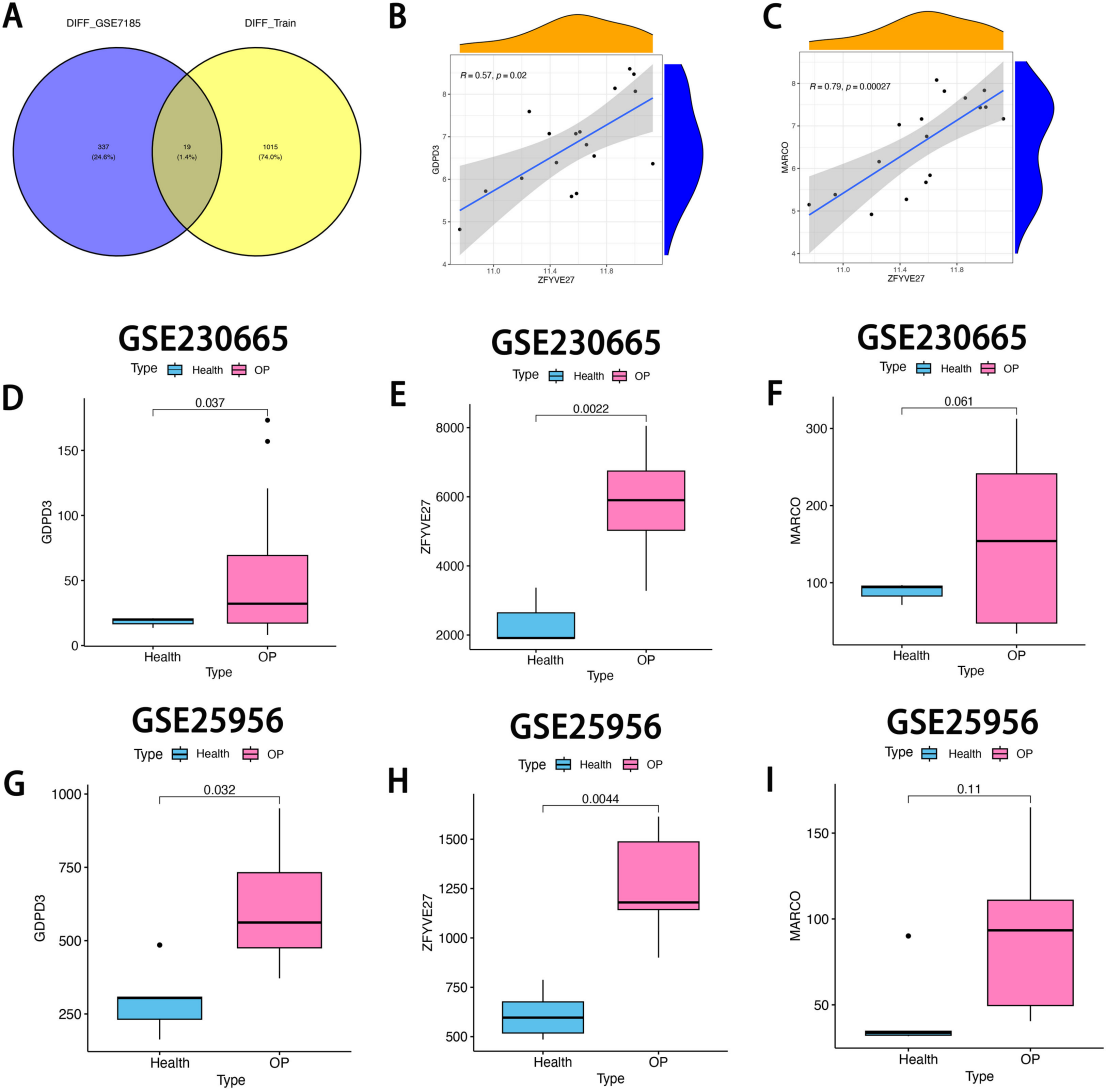
Potential interaction molecules of ZFYVE27 and expression analysis in test sets. **(A)** Intersection of differential genes for ZFYVE27 in the validation and test sets. **(B)** Correlation between ZFYVE27 and GDPD3. **(C)** Correlation between ZFYVE27 and MARCO. **(D–F)** Differential expression of ZFYVE27, GDPD3, and MARCO in GSE230665. **(G–I)** Differential expression of ZFYVE27, GDPD3, and MARCO in GSE35956. GDPD3, glycerophosphodiester phosphodiesterase domain containing 3; MARCO, macrophage receptor with collagenous structure.

An additional expression matrix for OP was obtained from GSE7185. After grouping based on ZFYVE27, it was observed that, both in our study samples and in the GSE7185 expression matrix, GSEA (**Supplementary Figures 3A, B**), KEGG analysis (**Supplementary Figures 3C, D**), and GO analysis (**Supplementary Figures 3E, F**) all showed enrichment in the same pathways, consistent with our previous research. This demonstrates the stability of ZFYVE27 as a disease diagnostic marker. To identify interaction partners of ZFYVE27, differential analysis was performed on the dataset (**Supplementary Figures 3G, H**) and 19 related genes were identified (**Figure 5A**). Correlation analysis showed that ZFYVE27 exhibited high correlation with GDPD3 (R = 0.57) and MARCO (R = 0.79) (**Figures 5B, C**). Furthermore, the expression levels of these three genes were consistently up-regulated in OP patients from both GSE230663 and GSE25956 (**Figures 5D–I**).

### 3.7 Expression levels of ZFYVE27 in an OP model

**Figure 6:**
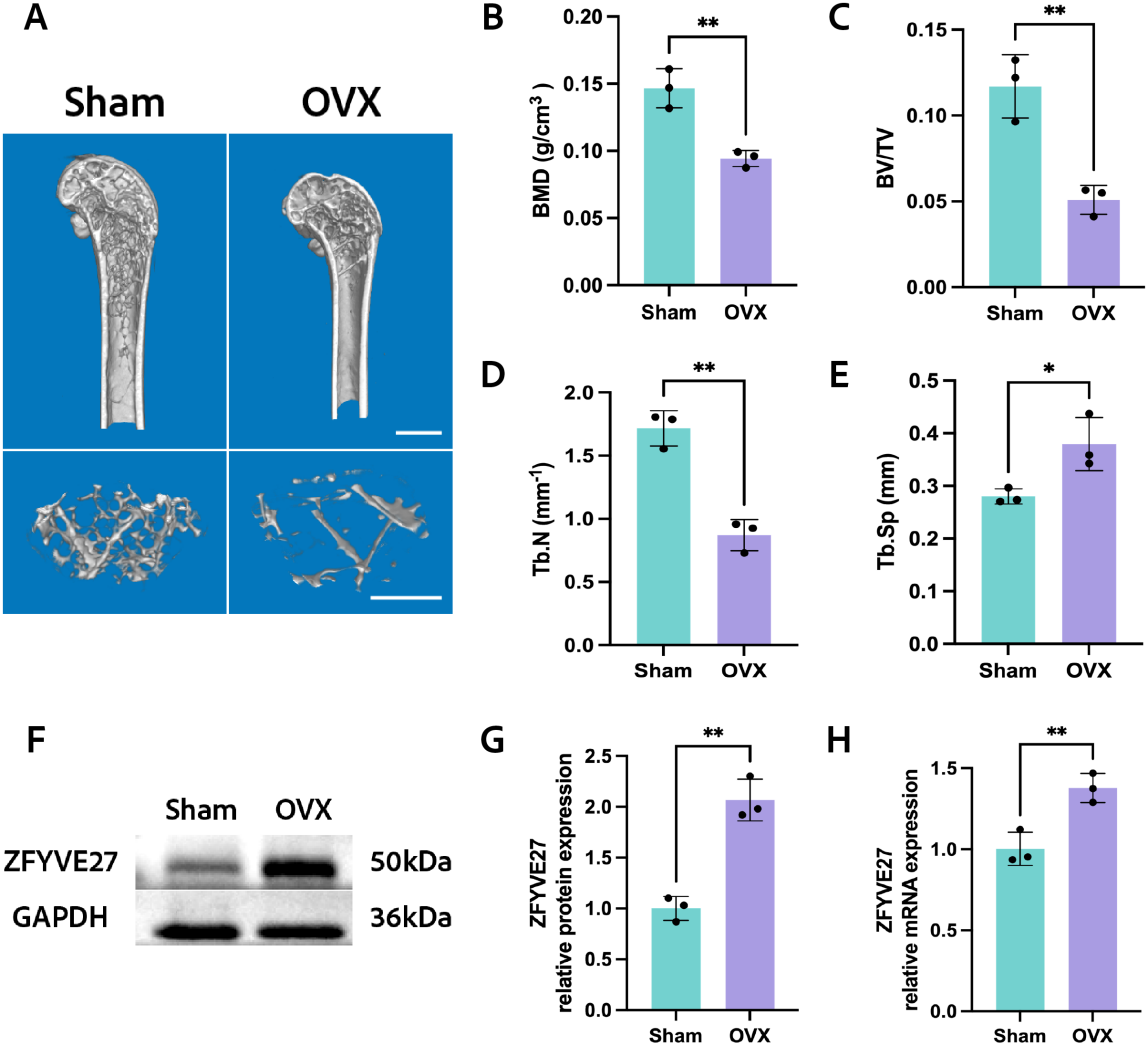
Expression levels of ZFYVE27 in an osteoporosis model. **(A)** Micro-CT images of the distal femur from 8-week-old female Sham and OVX mice. Scale bars, 1 mm. **(B–E)** Quantification of BMD, BV/TV, Tb.N, and Tb.Sp (n = 3 biological replicates). **(F)** The mRNA expression levels of ZFYVE27 were assessed in Sham and OVX groups using RT-qPCR analysis. **(G, H)** The protein expression levels of ZFYVE27 were assessed in Sham and OVX groups using Western blotting analysis. Data are presented as mean ± SD (*p < 0.05, **p < 0.01). Micro-CT, micro-computed tomography; OVX, ovariectomized; BMD, bone mineral density; BV/TV, trabecular bone volume per total volume; Tb.N, trabecular number; Tb.Sp, trabecular separation; RT-qPCR, reverse transcription quantitative polymerase chain reaction.

In this study, the OP model was induced by OVX in the 8-week-old C57BL/6J female mice. BMD, BV/TV, Tb.N and Tb.Sp were measured using micro-CT. Micro-CT evaluation revealed a significant reduction in BMD, BV/TV, and Tb.N, alongside a concomitant increase in Tb.Sp in the OVX group relative to the sham controls (*p* < 0.05) (**Figures 6A–E**). Thereafter, western blotting and RT-qPCR assays were performed using femur samples from mice in the sham and OVX groups. The results showed that the protein and mRNA levels of ZFYVE27 were significantly higher (*p* < 0.01) in the OVX group than in the sham group (**Figures 6F–H**).

### 3.8 Construction of the ceRNA network

According to the aforementioned screening criteria, only miRNA (miR-1207-3p) associated with ZFYVE27 were identified. SpongeScan-predicted lncRNA-miRNA interactions were used to construct a ZFYVE27-centered ceRNA network using Cytoscape (**Supplementary Figure 4**).

## 4 Discussion

OP is a multifactorial skeletal disorder, with estrogen deficiency serving as a primary pathogenic driver. This deficiency can trigger low-grade inflammation, cellular senescence, and immune remodeling (8, 9). This study integrated bioinformatics and machine learning to identify OP-associated gene modules via WGCNA and validated key genes through multi-algorithm screening. The constructed ZFYVE27-targeted ceRNA network revealed novel post-transcriptional regulatory insights. These findings advance the understanding of OP pathogenesis, offering potential diagnostic biomarkers and therapeutic targets for early intervention.

Differential gene expression analysis revealed significant alterations in OP patients compared to healthy individuals, implicating key pathways such as FAs, PTH signaling, protein metabolism, and adrenergic signaling. FAs, critical structures mediating cell-extracellular matrix interactions, regulate bone cell motility, differentiation, and signaling. Kindlin-2 gene mutations disrupt FAs function, impairing bone homeostasis (10). In OP, upregulated FAs may exacerbate osteoclast hyperactivity or osteoblast dysfunction. PTH can increase the activity of osteoclasts and promote bone resorption, while stimulating osteoblasts to form new bone (11). However, long-term high levels of PTH can also promote bone resorption and aggravate the development of OP (12). Therefore, regulating PTH levels is one of the important strategies for the treatment of OP. Protein digestion/absorption pathways are implicated in bone matrix maintenance, suggesting that OP may trigger adaptive increases in protein metabolism to support bone repair (13). Adrenergic signaling influences bone remodeling, with chronic sympathetic activation (e.g., in heart failure) linked to bone loss (14). β-adrenergic blockers counter this by promoting bone formation (15). Calcium/CAM signaling, mediated by calcium-binding proteins, regulates cell differentiation through intracellular calcium dynamics (16). Small GTPases, particularly RhoA, regulate cytoskeletal organization and vesicle transport in bone cells (17). RhoA activation via the Akt-mTOR-NFATc1 pathway enhances osteoclast activity, thereby driving OP progression (18). Targeting RhoA or its downstream effectors could mitigate pathological bone resorption. These findings highlight the multifactorial mechanisms underlying OP pathogenesis. Therapeutic strategies may include balancing PTH levels, suppressing sympathetic hyperactivity, modulating calcium-CAM pathways, or targeting RhoA to restore bone homeostasis.

The WGCNA tool was utilized to identify the gene modules highly correlated with OP, and the key module genes were extracted. Consistent with our findings, previous studies have established that metabolic perturbations, including lipid metabolism dysregulation and familial hyperlipidemia, severely compromise bone homeostasis. Studies have reported that abnormal lipid metabolism can lead to bone cell dysfunction, affect bone structure and function, and increase the risk of OP (19). Similarly, glycerin metabolism, glycerophospholipid metabolism and nitrogen metabolism may affect the occurrence and development of OP via various mechanisms, consistent with previous findings (20). In addition, there are common risk factors between cardiovascular and musculoskeletal diseases, like smoking, alcohol consumption, and menopause, and vascular calcification and the release of inflammatory factors may be a bridge between the two. These genes are not only closely related to the pathological process of OP, but are also expected to be the potential therapeutic targets.

Immune cell correlation analysis revealed significant differences in immune cell profiles between healthy and OP samples. Notably, seven core genes demonstrated close associations with distinct immune cell types. Estrogen deficiency in postmenopausal women was found to dysregulate CD4^+^ T cells, leading to elevated levels of inflammatory cytokines (TNF-α, IFN-γ, IL-17, RANKL, and CD40L) that disrupt bone homeostasis, which may contribute to the pathogenesis of PMOP (1). Zhu et al. (21). reported that a decrease in the number or function of CD4^+^ and CD8^+^ regulatory T cells may lead to increased bone loss, thereby triggering osteoporosis. Furthermore, estrogen deficiency disrupts the balance between Th17 and Treg cells, leading to an increase in Th17 cells and a decrease in Treg cells. This imbalance arises from both the impaired immunosuppressive function of Treg cells and their conversion into Th17 cells, which mechanistically explains the Th17/Treg dysregulation observed in PMOP (22). B cells differentiate into antibody-producing plasma cells after antigen stimulation. PMOP patients exhibit reduced bone marrow B cells compared to healthy individuals, a finding consistent with (23). In the B-cell knockout mice, their bone marrow lacks Osteoprotegerin, and OP is more pronounced than that in the control group (24). Sapra et al. (25). found that Bregs have the potential to inhibit RANKL-induced osteoclastogenesis in a dose-dependent manner. Therefore, there are still many mysteries about the estrogen-T/B cell-bone cell axis, and further studies are required to clarify the molecular mechanisms.

Macrophages represent pivotal orchestrators of bone homeostasis. The pro-inflammatory M1 phenotype exhibits osteoclastogenic potential, whereas the anti-inflammatory M2 macrophages are conventionally recognized for their osteogenic contributions. Under OP conditions, macrophages mainly exhibit the pro-inflammatory M1 phenotype and release inflammatory cytokines including IL-1, IL-6 and IL-17, which are closely associated with bone absorption and the increased osteoclast activity (26). On the other hand, in the microenvironment that promotes M2 polarization, cytokines secreted by macrophages including IL-4, IL-10, transforming growth factor β, and bone morphogenetic protein 2 facilitate osteoblast proliferation and mineralization (27). In addition, in a mouse model of OVX-induced OP, researchers find that estrogen deficiency induces the differentiation of M2 macrophages into functional osteoclasts, and estrogen therapy reverses this phenomenon (28). All in all, the influence of macrophages on osteocytes may be attributed to their different polarization states and the paracrine factors that they secrete. Therefore, the role of macrophages in OP needs to be further explored. In the case of estrogen deficiency, the survival time of dendritic cells is significantly extended, accompanied by the increased expression of IL-7 and IL-15, which further promotes the production of IL-17 and TNF-α in memory T cell subsets. These inflammatory cytokines cause bone loss associated with PMOP inflammation through inducing low-grade inflammation (29). Besides, estrogen deficiency may result in overactivation of neutrophils, cause osteoblast apoptosis through the release of reactive oxygen species, and increase osteoclast generation via the RANKL signaling pathway (22). Therefore, estrogen and immune cells are jointly involved in the occurrence of PMOP.

Additionally, KEGG analysis suggested that core genes were significantly associated with the Wnt pathway and Notch signaling pathway. The Wnt/β-catenin pathway drives osteoblast differentiation and is a key therapeutic target for OP, with activators enhancing bone formation. The Wnt ligand binds LRP5/6 and Frizzled receptors, triggering signaling that activates β-catenin, which accumulates, translocates to the nucleus, and promotes osteoblast target gene transcription (30). Moreover, the Notch signaling pathway executes different functions in osteoblasts, osteocyte, osteoclasts, and bone marrow-derived mesenchymal stem cells. Specifically, activation of Notch signaling in immature osteoblasts causes impaired osteoblast differentiation, resulting in reduced bone volume, whereas its activation in osteocyte inhibits initial bone resorption and increases bone volume (31). Activating the Notch signaling pathway in mesenchymal stem cells can up-regulate osteoblast-related genes and down-regulate osteoclast-related genes, therefore restoring the bone metabolic balance (32). Thus, the Notch signaling pathway has a dual effect on bone turnover depending on the cellular environment.

We further employed the RF and SVM algorithms to filter out the differentially-expressed genes related to the disease. Ultimately, ZFYVE27 was determined to be the potential diagnostic biomarker for OP. To further verify the reliability of ZFYVE27, the OVX-induced OP model was constructed. The occurrence of OP was confirmed by micro-CT in the OVX group. The femur samples from the Sham group and OVX group were analyzed by RT-qPCR and Western blotting assays. The results indicated that when compared with the Sham group, the ZFYVE27 mRNA and protein expression levels significantly increased in the OVX group. Notably, the consistent upregulation of ZFYVE27 in both human OP transcriptomic datasets and the OVX mouse pathological model reveals remarkable cross-species expression consistency. This consistency not only validates the reliability of our bioinformatics screening but also indicates that ZFYVE27 exerts a conserved and pivotal biological role in the pathogenesis of OP. Accordingly, it greatly strengthens the clinical translational potential of ZFYVE27, supporting its candidacy as a promising and reliable biomarker for the diagnosis and targeted therapy of PMOP. ZFYVE27 (also known as Protrudin) is a protein containing the FYVE domain, which is mainly involved in intracellular transport, axon growth and cell signal transduction (33). At present, there are few studies on the direct association of ZFYVE27 with the skeletal system, but based on its known function, it may participate in bone metabolism or bone diseases. ZFYVE27 exerts a dynamic effect on the membrane contact sites between endoplasmic reticulum, endosome and lysosome, and may affect the secretion function of osteoblasts or osteoclasts by regulating intracellular vesicle transport. For instance, the secretion of bone matrix proteins (such as collagen and osteocalcin) by osteoblasts depends on the vesicular transport system, and ZFYVE27 may be involved in this process. Moreover, the bone resorption function of osteoclasts relies on the release of lysosomal enzymes, and ZFYVE27 may affect its activity through regulating the related vesicle transport (34). ZFYVE27 is highly expressed in neurons, and as the nervous system is closely related to bone metabolism (e.g., sympathetic regulation of bone remodeling), ZFYVE27 may indirectly affect bone homeostasis through neural signaling (35). Furthermore, ZFYVE27 regulates autophagy by promoting the autophagosome-lysosome fusion, while autophagy is essential for the survival and function of osteoblasts and osteoclasts (36).

Glycerophosphodiester phosphodiesterase domain Containing 3 (GDPD3) belonging to the glycerol phosphodiesterase family, is involved in lipid metabolism, particularly the decomposition of lysophosphatidic acid (37). At present, there are few direct studies on GDPD3 and OP. GDPD3 exerts a vital role in phospholipid metabolism, and we speculate that GDPD3 may regulate bone metabolism through affecting the pathways related to phospholipid metabolism. Li et al. (38). reported that an increase in GDPD3 can inhibit the transformation of macrophages from M1 to M2 phenotype. Therefore, GDPD3 may participate in the occurrence of OP by regulating macrophage polarization.

Macrophage receptor with collagenous structure (MARCO) is a scavenger receptor highly expressed on macrophages and dendritic cells (39). The relationship between MARCO and OP is still in the preliminary research stage, but based on its biological function and the role of macrophages in bone metabolism, it is hypothesized that MARCO may affect the differentiation of macrophages into the proinflammatory (M1) phenotype and release proinflammatory factors to disrupt bone homeostasis. Besides, MARCO participates in the uptake of oxidized low-density lipoprotein, while lipid metabolic abnormalities often co-exist with OP, suggesting a potential common pathological mechanism (such as oxidative stress).

In addition, lncRNA, miRNA and mRNA can form the ceRNA regulatory networks to participate in the occurrence of OP (40). The potential ceRNA regulatory network targeting ZFYVE27 was constructed in this study, offering a new reference for exploring the regulatory mechanisms and targeted therapy of OP. Notably, in the ceRNA network constructed in this study, KCNQ1OT1 was identified as a potentially key lncRNA associated with ZFYVE27, suggesting that it may play a regulatory role in the development and progression of OP. As reported by Yang et al. (41), lncRNA KCNQ1OT1 up-regulated the expression of RICTOR by inhibiting miR-205-5p in mesenchymal stem cells, thus promoting osteogenic differentiation. Wang et al. (42) found that lncRNA KCNQ1OT1 promotes osteogenic differentiation by inhibiting miR-98-5p and upregulating Tbx5 expression, suggesting that this axis may represent a potential therapeutic target for osteoporosis. Therefore, LncRNA KCNQ1OT1 may be a candidate gene for treating OP. Although direct experimental evidence regarding miR-1207-3p in bone metabolism remains unreported, previous studies have highlighted its crucial regulatory roles in cellular proliferation and apoptosis in various disease models (43, 44). The progression of osteoporosis is fundamentally driven by an imbalance in the proliferation and apoptosis of osteoblasts and osteoclasts. Therefore, we reasonably hypothesize that the identified miR-1207-3p/ZFYVE27 axis might influence bone homeostasis by modulating the proliferative and apoptotic capacities of local bone-related cells, which warrants further experimental validation in the future.

The results of the present study demonstrate that ZFYVE27 and its related genes play a critical role in the pathogenesis of OP, as validated by both bioinformatic datasets and *in vivo* experiments. Nonetheless, some limitations should be noted in this study. At first, the sample size of gene expression profiles obtained from public databases was relatively small, and individual differences between samples might affect the universality of results. Second, although we used cross-validation in model training, the relatively small sample size may still lead to overfitting, thereby affecting the robustness and generalizability of our results. Therefore, future studies with larger cohorts are warranted to mitigate this risk and further validate the accuracy of the model’s predictions. Third, the ceRNA regulatory network obtained based on ZFYVE27 remains in the preliminary research stage, which requires validation by further *in vitro* and *in vivo* experiments. Furthermore, although the OVX mouse is a well-recognized and widely used model for PMOP, there are inherent biological and physiological differences between murine models and humans. Specifically, several key discrepancies should be noted: (1) Estrogen levels drop rapidly after OVX in mice, whereas PMOP in humans is characterized by a gradual and progressive decline in estrogen; (2) OVX mice exhibit an initial acute inflammatory response followed by low-grade chronic inflammation, which differs from the persistent, low-grade chronic inflammation associated with gradual estrogen depletion in humans; (3) OVX models are typically established using 8-to-12-week-old mice, which lack the pathological changes associated with aging and immunosenescence commonly observed in PMOP patients. Therefore, while our *in vivo* findings provide preliminary mechanistic insights, larger and well-designed clinical cohorts are warranted to validate our results and further evaluate their clinical translational potential. Furthermore, the public datasets and the 2-month post-OVX mouse model utilized in this study primarily represent an established state of OP. Whether ZFYVE27 expression becomes dysregulated prior to significant bone mass loss, thereby holding genuine early warning potential, remains to be determined. Future longitudinal and time-course studies are urgently needed to rigorously evaluate its predictive and diagnostic value for early-stage OP.

## 5 Conclusion

This study elucidates the pivotal roles of ZFYVE27, GDPD3, and MARCO in the pathogenesis of osteoporosis and ultimately pinpoints ZFYVE27 as a potential biomarker for this disease. Future studies are needed to further validate how the core genes affect bone homeostasis through lipid metabolism or immune system pathways. These validations will contribute to a better understanding of the pathophysiological mechanisms of OP and help improve the clinical diagnosis and therapeutic intervention of OP.

## Data Availability

The original contributions presented in the study are included in the article/**Supplementary Material**. Further inquiries can be directed to the corresponding author.
