# Supplementary material for: Screening and validation of ZFYVE27 as a potential diagnostic biomarker for osteoporosis via integrative bioinformatics and machine learning approaches
Source: Front Immunol. 2026 Jun 24;17:1862140. doi: 10.3389/fimmu.2026.1862140 (PMC13374800; doi:10.3389/fimmu.2026.1862140)
Supplement: Supplementary file 1 [file SupplementaryFile1.docx]

**Supplementary Material**

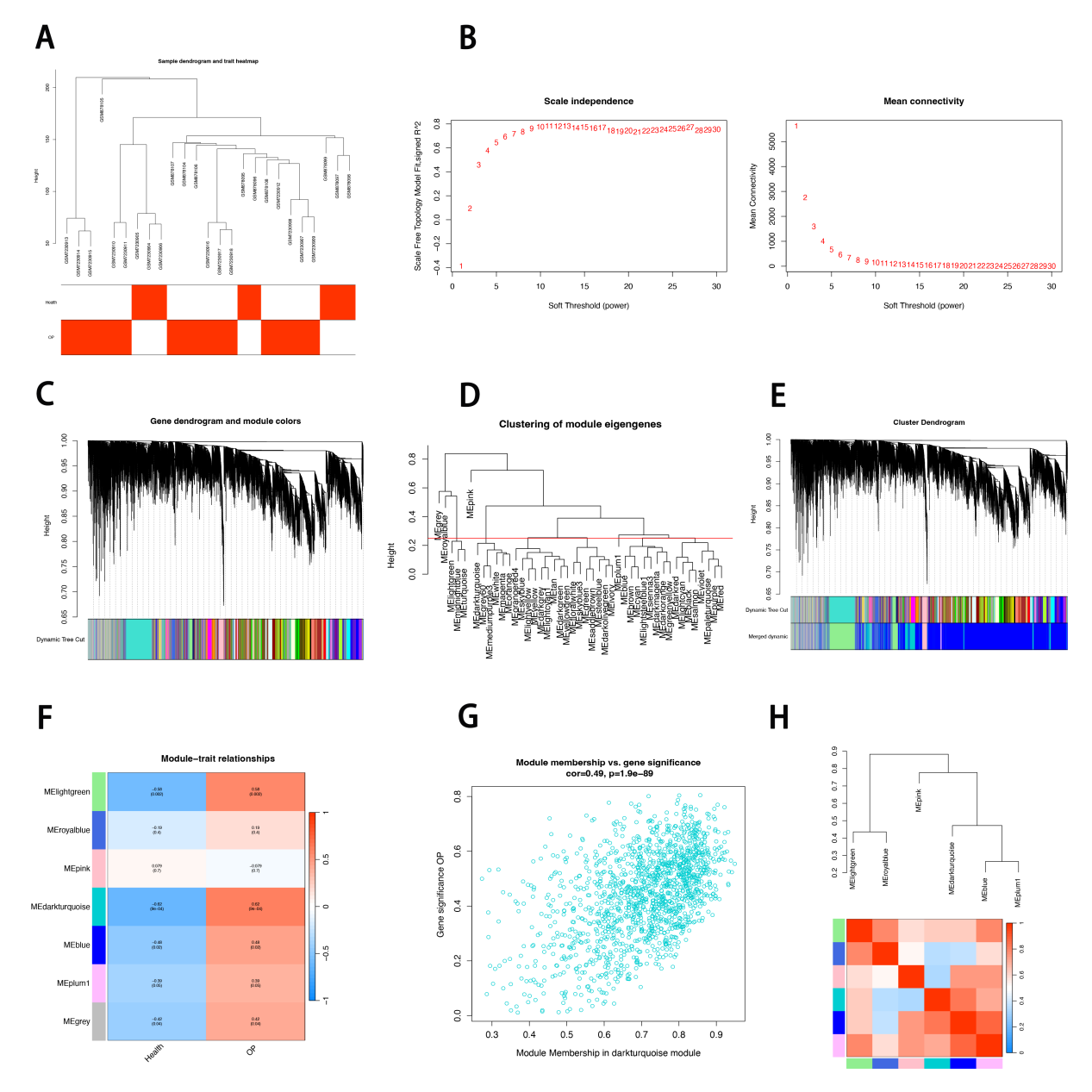


**Supplementary Figure 1** WCGNA analysis was performed to obtain disease-associated genes. **(A, B)** Confirmation of the optimal scale-free topology index under various soft thresholding powers (β), and the mean connectivity under different soft thresholding powers. **(C)** Gene tree diagram with node colors. **(D)** Hierarchical clustering analysis. **(E)** Cluster-based gene dendrogram. **(F)** Heatmap of module gene correlations with the two clusters. **(G)** Correlation of gene sets with OP. **(H)** Heatmap of all genes. OP, osteoporosis.

**
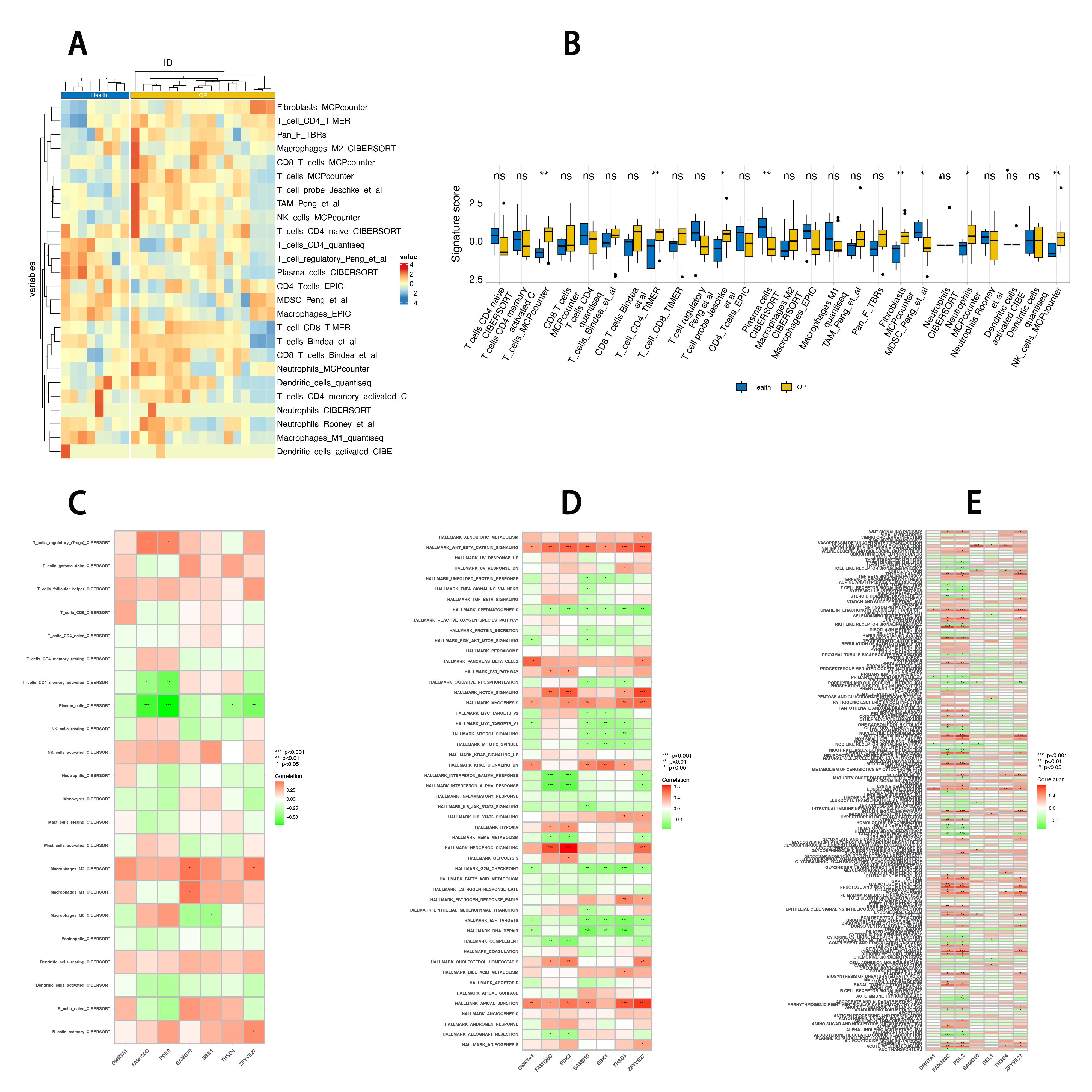
**

**Supplementary Figure 2** Immune and molecular functional landscapes. **(A, B)** Abundances of immune cell types in the healthy and OP groups. **(C)** Heatmap of scores for key genes based on LASSO in 22 immune-related genes. **(D)** Heatmap of scores for key genes based on LASSO in 50 hallmark gene sets. **(E)** Heatmap of scores for key genes based on LASSO in C2-KEGG pathways.

**
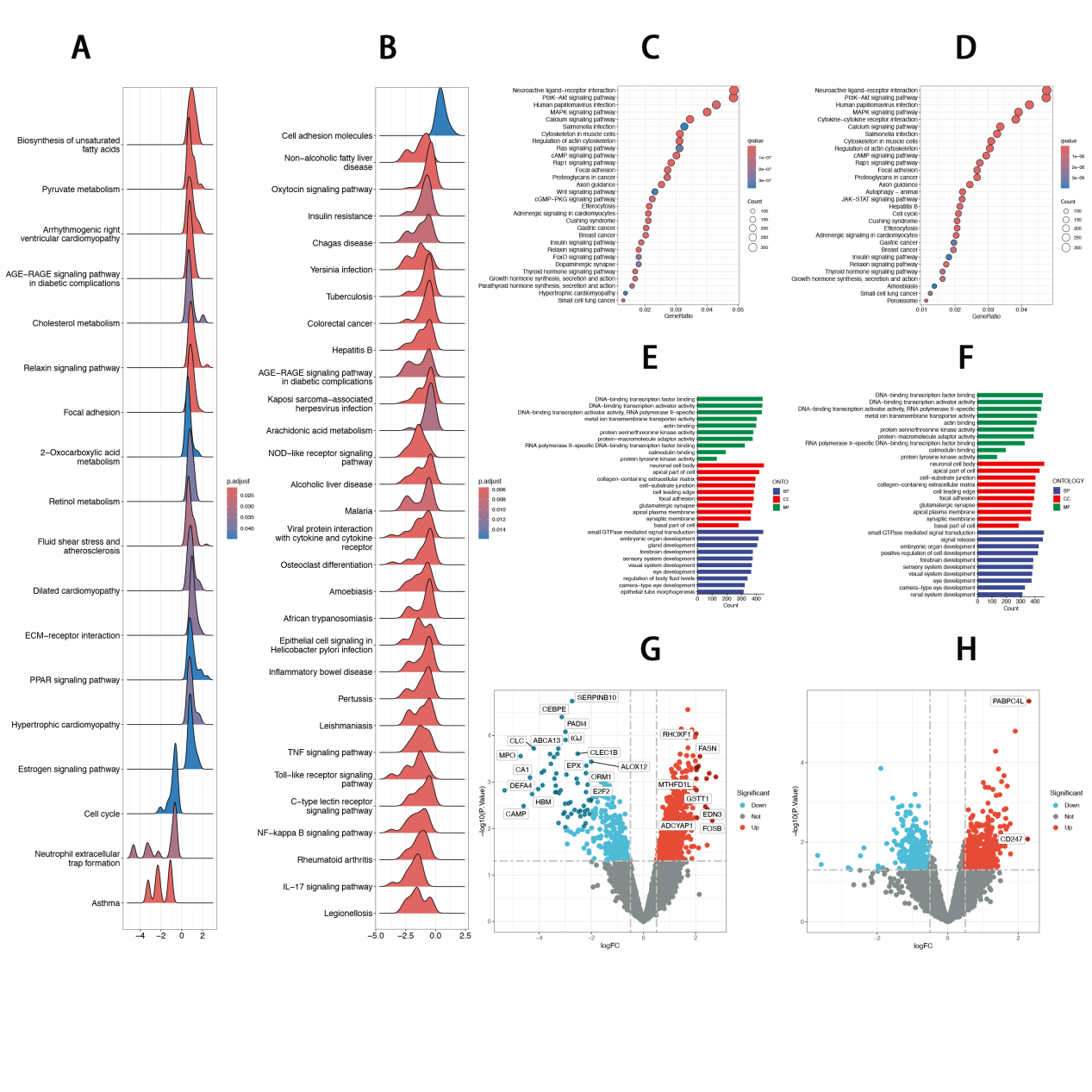

Supplementary Figure 3** Functional enrichment analysis of the ZFYVE27 gene in OP. **(A)** GSEA results for ZFYVE27 based on this study. **(B)** GSEA results for ZFYVE27 based on the GSE7185 dataset. (**C)** KEGG analysis results for ZFYVE27 based on this study. **(D)** KEGG analysis results for ZFYVE27 based on the GSE7185 dataset. **(E)** GO analysis results for ZFYVE27 based on this study. **(F)** GO analysis results for ZFYVE27 based on the GSE7185 dataset. **(G)** Differential volcano plot for ZFYVE27 based on this study. **(H)** Differential volcano plot for ZFYVE27 based on the GSE7185 dataset.


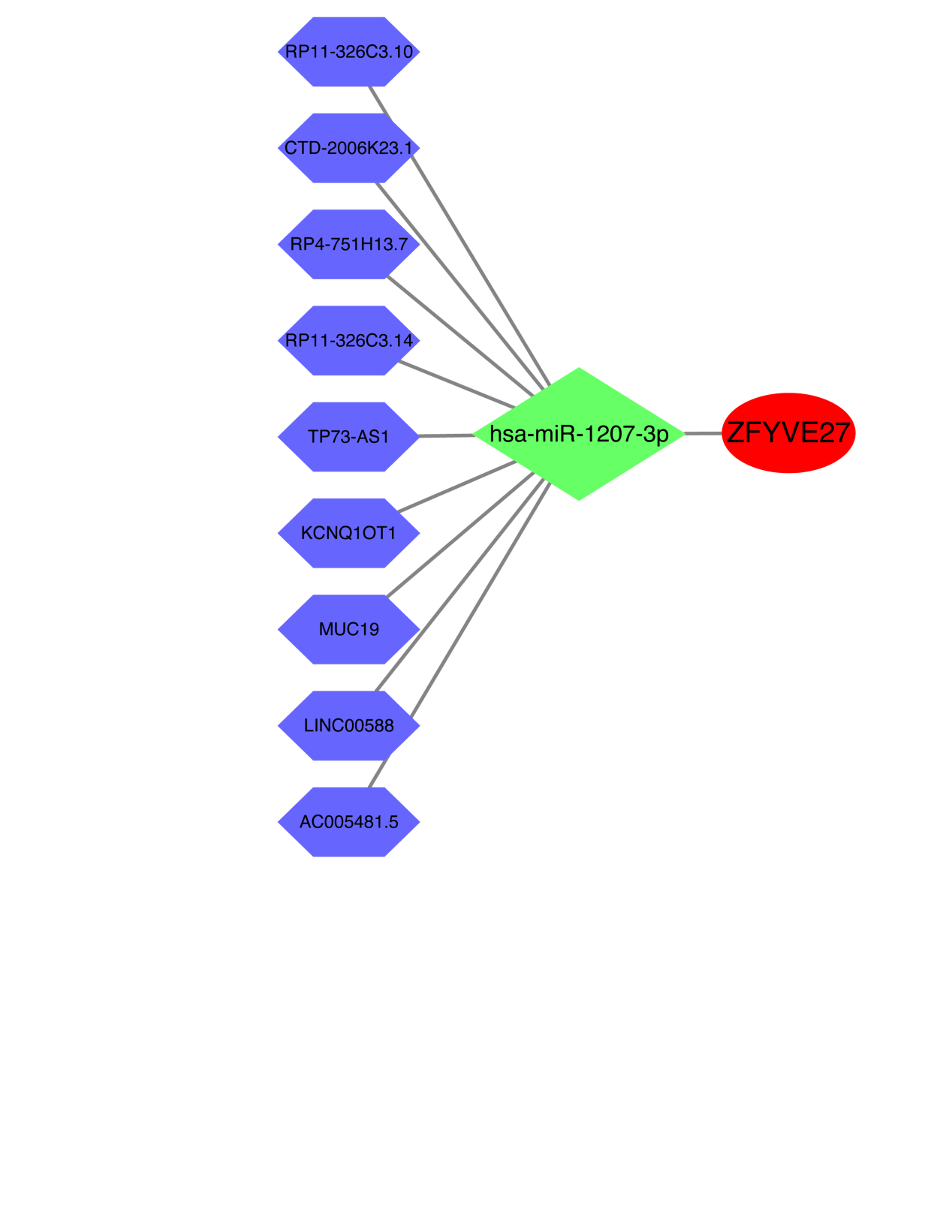


**Supplementary Figure 4** Construction of the ceRNA network. In the ceRNA network, red ovals represent core genes, green diamonds represent miRNAs, and blue hexagons represent lncRNAs. ceRNA, competitive endogenous RNA; lncRNA, long non-coding RNA.
