# Supplementary material for: Screening and validation of ZFYVE27 as a potential diagnostic biomarker for osteoporosis via integrative bioinformatics and machine learning approaches
Source: Front Immunol. 2026 Jun 24;17:1862140. doi: 10.3389/fimmu.2026.1862140 (PMC13374800; doi:10.3389/fimmu.2026.1862140)
Supplement: Supplementary file 2 [file Table1.docx]

**Supplementary Table 1** Primer sequences used in this study.

| **Gene** | **Stream** | **Sequence** |
| --- | --- | --- |
| Mouse GAPDH | Forward  Reverse | 5’-GGAGAGTGTTTCCTCGTCCC-3’  5’-ATGAAGGGGTCGTTGATGGC-3’ |
| Mouse ZFYVE27 | Forward  Reverse | 5’-AGATCCCAACAGAAGACCAAAGA-3’  5’-TCTTCATGCTTGGTACCCGATTT-3’ |
